# Prevalence and risk factors of low back pain among Lithuanian national defense volunteer forces

**DOI:** 10.1186/s12891-025-09235-1

**Published:** 2025-10-17

**Authors:** Vilma Dudonienė, Evaldas Žemaitis

**Affiliations:** Department of Health Promotion and Rehabilitation, Lithuanian Sports University, Kaunas, Lithuania

**Keywords:** Low back pain, Military, Prevalence, Risk factors

## Abstract

**Background:**

Low back pain (LBP) is a prevalent musculoskeletal disorder that imposes a significant burden on individuals and healthcare systems worldwide. Military personnel, including active and reserve forces, are particularly vulnerable to LBP due to the physically demanding nature of their duties, exposure to extreme conditions, and repetitive strain on the lower back. This study aimed to assess the prevalence of LBP and identify associated risk factors among members of the Lithuanian National Defense Volunteer Forces (LNDVF).

**Methods:**

A cross-sectional study was conducted with 272 active-duty LNDVF personnel (221 males and 51 females). A structured questionnaire collected data on demographics, lifestyle habits, health history, and LBP occurrence. LBP prevalence was assessed based on self-reported instances: lifetime prevalence, 12-month prevalence, and pain at the time of the survey. The analysis explored associations between LBP and potential risk factors, including gender, age, marital status, smoking, body mass index (BMI), history of back injuries, stress levels, and self-perceived health status.

**Results:**

The lifetime prevalence of LBP was 85.3%, the 12-month prevalence was 81.6%, and 42.3% of participants reported experiencing LBP at the time of the survey. Female soldiers reported a higher prevalence of LBP than males. Older soldiers (≥ 33 years) and unmarried personnel had a greater likelihood of experiencing LBP. Smokers and individuals with a higher BMI exhibited an increased risk, likely due to biomechanical strain. Elevated stress levels were positively correlated with LBP prevalence, emphasizing the link between psychological and physical health. A history of back injuries significantly increased the odds of experiencing LBP. Soldiers who rated their health as poor or average were more likely to report LBP.

**Conclusions:**

More than four-fifths of military personnel have experienced low back pain at least once in their lifetime and within the past 12 months, with nearly half reporting LBP at the time of the survey. Identified risk factors for LBP include female sex, older age, lack of a partner, previous back injuries, elevated stress levels, smoking, self-reported poor health, and higher body mass index. LBP significantly affects military readiness, impairing the ability to perform service tasks in approximately two-thirds of affected soldiers.

**Supplementary Information:**

The online version contains supplementary material available at 10.1186/s12891-025-09235-1.

## Background

Low back pain (LBP) is a leading cause of disability globally and imposes a significant economic burden on healthcare systems and society [1]. This condition is a common contributor to workplace absenteeism, further compounding its societal impact [2]. Estimates suggest that the prevalence of LBP in the adult population ranges from 50% to 70% [3]. Although LBP is widely recognized as a multifactorial disorder, pinpointing its exact etiology remains challenging due to the interplay of various physiological, psychological, and environmental factors [4]. Nevertheless, several well-documented risk factors have been consistently associated with the development of LBP, including psychological stress, obesity, advancing age, smoking, and a history of lower back injuries [5, 6]. These insights underscore the importance of targeted prevention and management strategies to mitigate the burden of LBP.

The military profession is inherently demanding, requiring personnel to engage in complex, high-intensity activities that test both physical and mental resilience. To maintain optimal performance and enhance physical fitness, military personnel undergo rigorous training regimens and operate in challenging environments [7]. These activities, which often involve prolonged physical exertion, heavy load-bearing, and repetitive motions, are crucial for mission success. However, they also increase the risk of musculoskeletal injuries, particularly LBP. Soldiers are frequently required to wear heavy tactical equipment, imposing significant strain on their musculoskeletal systems, especially the spine [5]. Identified occupational risk factors for LBP include heavy workloads, frequent heavy lifting, and repetitive bending [8]. A 2021 meta-analysis concluded that, among active-duty personnel, a prior history of LBP, previous musculoskeletal injury, reduced time spent in physical training, female sex, and lower military rank were consistent risk factors for LBP [9]. Furthermore, unspecified LBP experienced during military service has been established as a predictor of LBP in later life [10].

Globally, research highlights a high prevalence of LBP among military populations. Studies have reported acute LBP prevalence rates ranging from 61% to 84% among soldiers [11]. In Malaysia, the prevalence of LBP among soldiers was 48.9% [12], while in Saudi Arabia, it was 46.3% [13]. Among Chinese soldiers [14], it was found that 26.2% experienced LBP, with risk factors including night training, participation in a 5-km cross-country race, and grenade-throwing exercises. These findings underscore the importance of targeted prevention and intervention strategies [15, 16], and in some cases nonpharmacologic treatment modalities [17, 18] to reduce the impact of LBP in military settings.

Chan et al. [12] reported that soldiers with a history of lumbar trauma were 2.25 times more likely to experience low back pain (LBP) compared to those without such a history. Similarly, Monnier et al. [19] found that individuals who had experienced lumbar trauma were 2.99 times more likely to develop LBP within six months of the injury, with the risk increasing to 6.75 times within 12 months post-trauma, and that a history of lumbar injury may predispose individuals to musculoskeletal disorders by impairing muscle strength, joint stability, and proprioception. These deficits can compromise spinal biomechanics, disrupting the optimal distribution of mechanical loads on the spine and thereby elevating the risk of recurrent or chronic LBP [19]. In general, risk factors for low back pain (LBP) can be analyzed from physical, psychological, sociodemographic, and occupational perspectives [20].

Personnel of the Lithuanian National Defense Volunteer Forces (LNDVF) experience occupational demands similar to those reported by Yang et al. [8] and Perera et al. [6], with low back pain being recognized as a factor that hinders task performance. However, data on the prevalence of LBP within these forces and the contributing risk factors remain limited. Identifying these risk factors is crucial for developing targeted prevention and intervention strategies to mitigate the impact of LBP on military personnel. This study aims to assess the prevalence of LBP and identify its associated risk factors among LNDVF personnel.

## Methods

### Participants

Targeted sampling was employed to select the study participants, focusing on National Defense Volunteers. A total of 272 individuals (51 women and 221 men) with valid contracts with the National Defense Volunteer Forces on the day of the survey participated in the study.

The study sample was calculated with a margin of error of 5% using Paniotto’s formula: *n* = 1/(Δ2 + 1/N), where n represents the sample size, Δ is the permissible error (5%), N is the population size. For this study, the population size (N) was 900 (the 2nd territorial unit Darius and Girėnas district, consisting of 10 infantry companies with each company averaging 90 soldiers). Substituting the values into the formula: *n* = 1/(0.05²+1/900) = 269. Thus, the required sample size was determined to be 269 participants.

### Ethical approval

 The study was conducted between February and March 2024 among volunteers from the 2nd National Team of the Darius and Girėnas Districts. Ethical approval for the study was granted by the Lithuanian Sports University Bioethics Committee (Permit No. BNL-KIN (B)−2023-654, issued on January 17, 2024). All participants were informed in detail of the purpose of the study, and they signed an informed consent form. The study was conducted in accordance with the Declaration of Helsinki Ethical Principles. Written consent for the study was also obtained from the commander of the 2nd National Team of the Lithuanian National Defense Volunteer Forces (LNDVF).

### The survey

 The questionnaire survey was conducted using a mixed-method approach. The virtual version of the questionnaire was uploaded to designated social network groups used by volunteer soldiers for information sharing, while the paper version was distributed during training sessions. Volunteers were primarily informed about the study through verbal announcements during training sessions. Participation was voluntary, and no direct encouragement or pressure from commanding officers was applied.

The survey (see Supplement) consisted of 22 closed-ended questions [21] with predefined answer options and 4 additional closed-ended questions requiring participants to provide their age, height, weight, and years of military service. The questionnaire was structured into four categories: (1) Demographic Data: information on participants' gender, education, age, and marital status; (2) Health Characteristics: questions addressing factors classified as pain risk factors; (3) Professional Factors: aspects specific to the military service; (4) Low Back Pain: prevalence of LBP and its impact on military service.

### Statistical analysis

 Statistical analysis was conducted using the SPSS 29.0 software package. For the analysis of quantitative data, descriptive statistics were calculated, including the mean, standard deviation (SD), and median [25–75%]. For ordinal variables, the frequency (n) and percentage distribution were provided. The Kolmogorov-Smirnov test was applied to assess the normality of the data distribution. A confidence level of 95% (P = 0.95) and a significance level of 5% (p = 0.05) were used. The chi-square (χ²) test of independence was employed to examine the association between nominal variables or in frequency tables. Depending on the sample size, either exact tests (for small samples) or asymptotic methods were used. Binary logistic regression analysis was performed for prediction, with odds ratios (OR) and 95% confidence intervals (CI) reported.

## Results

### Demographic characteristics

 Table 1 presents the demographic characteristics of the study participants. A statistically significant majority were males (81.2%), while females comprised 18.8%. The largest proportion of participants were under 29 years old (48.9%), followed by those aged 30–39 years (37.9%), with a significantly smaller proportion being over 40 years old (13.2%). A substantial proportion of participants had higher education (43.4%) or secondary education (33.5%), while significantly fewer had vocational (19.1%) or primary education (4.0%). Marital status distribution showed nearly equal proportions of married (42.6%) and single (41.9%) participants, with smaller percentages being divorced (7.7%), widowed (0.7%), or preferring not to disclose their status (7.0%). In terms of BMI, more than half of the participants had a normal BMI (51.1%), while 35.7% were classified as overweight, and a significantly smaller proportion (13.2%) were obese (Table 1).

**Table 1:** Demographic data of the study participants (*n* = 272)

Criteria		Participants, *n*(%)	χ², df, *p* values
Gender	Male	221 (81.2)	χ²=106.25, df = 1, *p*< 0.001
	Female	51 (18.8)	
Age (years)	≤ 29	133 (48.9)	χ²=151.75, df = 3, *p* < 0.001
	30–39	103 (37.9)	
	≥ 40	36 (13.2)	
Education	Higher education	118 (43.4)	χ²=96.08, df = 3, *p* < 0.001
	Secondary education	91 (33.5)	
	Vocational education	52 (19.1)	
	Primary education	11 (4.0)	
Marital status	Married	116 (42.6)	χ²=1.74, df = 1, *p* = 0.187
	Single	114 (41.9)	
	Divorced	21 (7.7)	
	Widowed	2 (0.7)	
	Prefer not to answer	19 (7.0)	
BMI (kg/m²)	Normal	139 (51.1)	χ²=161.50, df = 3, *p* < 0.001
	Overweight	97 (35.7)	
	Obesity	36 (13.2)	

### Health characteristics

 Table 2 summarizes the health-related characteristics of the study participants. A significant majority (69.9%) reported experiencing stress, while 30.1% did not. Among those experiencing stress, 55.6% reported moderate stress, 28.0% reported low stress, and 16.4% reported high stress, with the distribution being statistically significant. Most participants (57.7%) rated their health as good, while a significantly smaller proportion (28.7%) rated it as average, an even smaller proportion (10.3%) as very good, and only a few (3.3%) as poor, with statistically significant differences among these categories. A significant proportion of participants (65.1%) reported never having experienced back injuries, while a smaller proportion (22.1%) had a history of back injuries, and some (12.9%) did not respond to this question (Table 2).

**Table 2:** Health characteristics of the study participants (*n* = 272)

Health Characteristics		Participants, *n* (%)	χ², df, *p* values
Experience stress	Yes	190 (69.9)	χ²=42.88, df = 1, *p* < 0.001
	No	82 (30.1)	
Perceived stress level	High	31 (16.4)	χ²=45.84, df = 2, *p* < 0.001
	Moderate	105 (55.60	
	Low	53 (28.0)	
Self-Assessed Health	Very good	28 (10.3)	χ²=192.67, df = 3, *p* < 0.001
	Good	157 (57.7)	
	Average	78 (28.7)	
	Poor	9 (3.3)	
Back injuries	Yes	60 (22.1)	χ²=57.75, df = 1, *p* < 0.001
	No	177 (65.1)	
	Did not respond	35 (12.9)	
Smoking	Yes	126 (46.3)	χ²=1.47, df = 1, *p* = 0.225
	No	146 (53.7)	

### Professional factors

 The professional characteristics of the study participants are presented in Table 3. A statistically significant majority (85.3%) of respondents were privates, while a smaller proportion were non-commissioned officers (12.5%) or officers (2.2%). Regarding service duration, the largest proportion of participants (45.2%) had served for 1–5 years, followed by those with 5–10 years of service (28.7%), less than 1 year (19.1%), and over 10 years (7.0%).

**Table 3:** Professional factors of the study participants (*n* = 272)

Professional Factors		Participants, *n* (%)	*p* value
Military status	Private	232 (85.3)	χ²=334.79, df = 2, *p* < 0.001
	Non- commissioned officer	34 (12.5)	
	Officer	6 (2.2)	
Duration of service (yrs.)	< 1	52 (19.1)	χ²=85.02, df = 3, *p* < 0.001
	1–5	123 (45.2)	
	5–10	78 (28.7)	
	> 10	19 (7)	

### Prevalence of low back pain

 It was found that 85.3% of study participants had experienced LBP at least once in their lifetime, 81.6% within the past 12 months, and 42.3% at the time of the study.

Table 4 presents the impact of LBP on military service among participants. A statistically significant majority (62.6%) reported that LBP limited their service opportunities, while a significantly smaller proportion (37.4%) indicated no such limitations. Regarding service absences, 28.7% of participants reported missing service due to LBP, whereas a statistically significant majority (71.3%) reported no absences. Additionally, 47.0% of participants believed their LBP was related to military service, while 53.0% did not, with the difference not reaching statistical significance (Table4).

**Table 4:** The impact of low back pain on military service

Criteria	Answer	Participants, *n* (%)	χ², df, *p* values
LBP limits service opportunities	Yes	72 (62.6)	χ²=7.31, df = 1, *p* = 0.007
	No	43 (37.4)	
Missed service due to LBP	Yes	33 (28.7)	χ²=20.87, df = 1, *p* < 0.001
	No	82 (71.3)	
LBP related to military service	Yes	54 (47.0)	χ²=0.426, df = 1, *p* = 0.514
	No	61 (53.0)	

### Relationship between low back pain and demographic characteristics

 Table 5 illustrates the relationship between the presence of LBP and various demographic characteristics. A chi-square test indicates a significant association between gender and LBP, with females being more likely to report LBP than males. No significant associations were found between age and LBP or between education level and LBP. However, single, divorced, or widowed individuals reported a significantly higher prevalence of LBP compared to married participants (Table 5).

**Table 5:** The relationship between low back pain and demographic characteristics

Demographic characteristics		Low Back Pain		χ², df, p values
		Yes n (%)	No n (%)	
Gender	Male	86 (38.9)	135 (61.1)	χ² = 5.47, df = 1, *p* < 0.019
	Female	29 (56.9)	22 (43.1)	
Age (years)	≤ 29	49 (36.8)	84 (63.2)	χ² = 3.71, df = 2, *p* = 0.156
	30–39	47 (45.6)	56 (54.4)	
	≥ 40	19 (52.8)	17 (47.2)	
Education	Higher education	48 (40.7)	70 (59.3)	χ² = 0.499, df = 3, *p* = 0.919
	Secondary education	38 (41.8)	53 (58.2)	
	Vocational education	24 (46.2)	28 (53.8)	
	Primary education	5 (45.5)	6 (54.5)	
Marital status	Married	40 (34.5)	76 (65.5)	χ² = 5.35, df = 1, *p* < 0.021
	Single, Divorced, Widowed	67 (48.9)	70 (51.1)	

Although no statistically significant difference was observed between age groups, the Receiver Operating Characteristic analysis identified a threshold age of 33 years as a predictor for back pain. The results indicate that respondents older than 33 years have an odds ratio of 1.875 [1.113–3.160] for experiencing back pain.

The relationship between LBP and health-related factors is presented in Table6. The chi-square test demonstrates a significant association between low back trauma and LBP. No significant association between BMI and LBP was found. A highly significant association between self-assessed health and LBP, with poorer self-assessed health strongly correlated with higher rates of LBP and a significant association between smoking and LBP is demonstrated. A significant association was found between experienced stress and LBP, as well as between a high perceived stress level and LBP.

**Table 6:** Relationship between low back pain and health related factors

Health related factors		Low Back Pain		χ², df, p values
		Yes, n (%)	No, n (%)	
Injuries of lower back	Yes	38 (63.3)	22 (36.7)	χ² = 7.05, df = 1, *p* < 0.008
	No	77 (43.5)	100 (56.5)	
BMI kg/m²	Normal	48 (35,5)	89 (65.5)	χ² = 5.03, df = 2, *p* = 0.081
	Overweight	49 (50.5)	48 (49.5)	
	Obese	16 (44.4)	20 (55.6)	
Self-Assessed Health	Very good	4 (14.3)	24 (85.7)	χ² = 34.98, df = 3, *p* < 0.001
	Good	55 (35.5)	102 (65.0)	
	Average	47 (60.3)	31 (39,7)	
	Poor	9 (100.0)	0 (0.0)	
Smoking	Yes	62 (49.2)	64 (50.8)	χ² = 4.61, df = 1, *p* < 0.032
	No	53 (36.3)	93 (63.7)	
Experienced stress	Yes	89 (46.8)	101 (53.2)	χ² = 5.37, df = 1, *p* < 0.020
	No	26 (31.7)	56 (68.3)	
Perceived stress level	High	24 (77.4)	7 (22.6)	χ² = 15.56, df = 2, *p* < 0.001
	Moderate	46 (43.8)	59 (56.2)	
	Low	18 (34.0)	35 (66.0)	

No statistically significant associations were observed between military status or duration of service and LBP prevalence (Table 7).

**Table 7:** The relationship between prevalence of low back pain and professional factors

Professional factors		Low back pain		χ², df, p values
		Yes, n (%)	No, n (%)	
Military status	Private	99 (42.7)	133 (57.3)	χ² = 0.10, df = 1, *p* = 0.752
	Non- commissioned officer, Officer	16 (40.0)	24 (60.0)	
Duration of Service (yrs.)	< 1	20 (38.5)	32 (61.5)	χ² = 7.06, df = 3, *p* = 0.070
	1–5	54 (43.9)	69 (56.1)	
	5–10	28 (35.9)	50 (64.1)	
	> 10	13 (68.4)	6 (31.6)	

Table 8 presents the final multivariable binary logistic regression model for predicting LBP in the participants, which includes predictors that were found to be significant in the univariate analysis. Based on the results, the likelihood of experiencing LBP is significantly higher in individuals who are female, have a BMI (kg/m²) greater than normal, are single, widowed, or divorced, and assess their health as average or poor.

**Table 8.** Multivariable binary logistic regression model for predicting lower back pain

**Analyzed characteristics (predictors)**	**Encountering lower back pain, Odds Ratio [95% Confidence Interval], p-value**
BMI (kg/m²)	1 1.909 [1.068–3.411.068.411], p = 0.029
Normal	
Overweight/Obesity	
Marital status	1 2.033 [1.139–3.627.139.627], p = 0.016
Married	
Single/Widowed/Divorced	
Subjective health assessment	1 3.136 [1.718–5.723.718.723], p < 0.001
Very good/Good	
Average/Poor	
Sex	1 2.292 [1.106–4.750.106.750], p = 0.026
Male	
Female	
Constant = −1.361, p < 0.001	

*OR* Odds Ratio, *CI* Confidence Interval, *p* Significance level

## Discussion

 The primary objective of this study was to assess the prevalence of back pain among National Defense Volunteer Forces personnel, while the secondary objective was to identify the risk factors associated with back pain. The findings revealed that 85.3% of participants reported having experienced LBP at least once in their lifetime, with 42.3% reporting current symptoms.

While these results appear consistent with prevalence rates reported in military samples from countries such as Malaysia [12] and Saudi Arabia [13], it is important to interpret such comparisons with caution. The physical demands, environmental conditions, healthcare systems, and training regimens differ substantially between countries. For instance, climatic conditions, operational tasks, and organizational structures in Malaysia and Saudi Arabia may differ significantly from those of the LNDVF. Therefore, although prevalence rates are similar, the underlying contributing factors and risk exposures may not be directly comparable.

This study also found that female sex was associated with a higher prevalence of low back pain. Similarly, Gun et al. [22] reported that female U.S. Army personnel were 66% more likely to experience back pain than their male counterparts. The authors suggest that this increased susceptibility may be attributed to lower average muscle mass, a higher percentage of body fat, and an elevated BMI among women compared to men. These factors may heighten the risk of repetitive strain injuries when exposed to similar physical loads in a military setting [22].

In our study, BMI was also identified as a significant risk factor for low back pain. Soldiers with a BMI above the normal range were significantly more likely to develop back pain. These findings align with the study by Gun et al. [22], which reported that overweight and obese soldiers were 77% more likely to experience low back pain. A higher BMI can negatively impact posture and alter movement kinematics, potentially increasing the risk of musculoskeletal injuries and back pain [23].

Additionally, this study found that current low back pain was more prevalent among soldiers with a prior history of back pain. Chan et al. [21] reported that soldiers who smoke are 1.7 times more likely to experience lower back pain compared to non-smokers. Similarly, our study found that 70% of smoking soldiers reported experiencing lower back pain more frequently. Smoking is associated with reduced blood flow, tissue hypoxia, and decreased bone density [24]. Furthermore, it can impair tissue healing and delay recovery [25]. Bedno et al. [24] also identified smoking as a significant risk factor for musculoskeletal injuries, which may contribute to the higher prevalence of lower back pain among military smokers.

This study also found that married soldiers were less likely to experience lower back pain. In contrast, Knox et al. [26] reported that single soldiers had a lower risk of low back pain. The authors attributed this to psychosocial factors but did not specify which factors contributed to the reduced risk [26]. In our study, the lower prevalence of low back pain among married soldiers may be explained by the social support available within a spousal relationship. Married individuals may remind each other to take medications, encourage physical activity, and provide emotional support, all of which contribute to overall health and may reduce the likelihood of experiencing lower back pain [27].

The results of this study indicate that soldiers experiencing stress are more likely to develop lower back pain. Furthermore, higher stress levels were significantly associated with an increased prevalence of this condition. Wei et al. [28] reported in their cohort study that soldiers who experienced psychological stress or strain were more likely to develop lower back pain, supporting the findings of our study. The authors suggest that this association may be explained by the interaction between pain and psychological factors, which can lead to behavioral and emotional changes that contribute to pain development [28].

Although a statistically significant difference in the prevalence of LBP across different age groups was not found, our data indicate that soldiers older than 33 years were more likely to experience LBP. Ernat et al. [29] reported a gradual increase in LBP prevalence with age among soldiers, noting that those aged 40 years and older had a one-third higher prevalence of LBP compared to soldiers under 20 years of age.

No statistically significant difference was observed in LBP prevalence based on military rank or length of service. However, some studies have suggested that lower-ranking soldiers experience LBP more frequently than senior officers [26]. This may be attributed to the fact that lower-ranking personnel often perform more physically demanding tasks, increasing their risk of musculoskeletal injuries, including LBP. In contrast, within the Lithuanian National Defence Forces, both soldiers and non-commissioned officers typically participate in training and operational tasks of similar physical intensity, which may explain the lack of a significant difference in LBP prevalence between these groups.

The survey revealed that more than two-thirds of soldiers reported that LBP limited their ability to perform professional duties, however more than half of the survey respondents with LBP did not attribute their condition to military service, possibly due to their concurrent employment in civilian occupations that may also contribute to musculoskeletal strain. Additionally, approximately one-third had missed at least one day of service in the past year due to LBP. These findings underscore the need to address modifiable risk factors and enhance soldier preparation to reduce LBP prevalence and ensure optimal operational readiness.

Understanding the prevalence and associated risk factors is essential for developing effective prevention strategies aimed at reducing LBP incidence or, when necessary, implementing appropriate treatment measures. Such initiatives would contribute to improving both the quality and efficiency of tasks performed by LNDVF personnel.

A key limitation of this study is that only one of the six national defense teams participated in the survey. Consequently, the findings may not fully reflect the overall prevalence and risk factors of LBP across the Lithuanian National Defense Forces. To obtain more comprehensive and generalizable results, future studies should include participants from all national defense teams. A broader sample would allow for a more accurate assessment of LBP prevalence and its associated risk factors across the entire forces. While stress was considered in this study, we acknowledge the limitation in the depth of psychological analysis. Future studies should incorporate a broader range of psychological variables (e.g., depression, anxiety, occupational burnout) and explore mediating pathways between mental health and pain development. This may also enable the use of more sophisticated statistical models, including interaction terms and mediation analysis, to better capture complex risk interrelationships.

Targeted interventions, including tailored exercise programs, stress management strategies, and smoking cessation initiatives, should be prioritized to mitigate LBP risk among military personnel. However, current evidence remains insufficient to provide clear guidance for military organizations and healthcare professionals in making informed treatment decisions for managing musculoskeletal pain in active-duty personnel [30]. Therefore, longitudinal studies are recommended to evaluate the effectiveness of these interventions and to explore the role of early detection and rehabilitation programs in reducing the burden of LBP within military settings.

## Conclusions

More than four-fifths of Lithuanian National Defense Volunteer Forces personnel have experienced low back pain (LBP) at least once in their lifetime and within the past 12 months, with nearly half reporting LBP at the time of the survey. Identified risk factors for LBP include female sex, older age, lack of a partner, previous back injuries, elevated stress levels, smoking, self-reported poor health, and higher body mass index. LBP significantly affects military readiness, impairing the ability to perform service tasks in approximately two-thirds of affected soldiers. Additionally, nearly one-third of personnel reported missing at least one day of service due to LBP in the past year.

Further research should focus on developing targeted prevention and intervention strategies to address these risk factors among military personnel. Longitudinal studies assessing the effectiveness of such interventions, along with the impact of early detection and rehabilitation programs, are necessary to reduce the prevalence of LBP and mitigate its consequences on military performance and overall health.

## Supplementary Information

Supplementary Material 1

## Data Availability

The datasets used and analysed during the current study are available from the corresponding author on reasonable request.

## Abbreviations

LBP: Low back pain
BMI: Body mass index
LNDVF: Lithuanian National Defense Volunteer Forces

## References

[1] Maher C, Underwood M, Buchbinder R. Non-specific low back pain. Lancet. 2017;389(10070):736–47. 27745712 10.1016/S0140-6736(16)30970-9

[2] Lardon A, Dubois J, Cantin V, Piché M, Descarreaux M. Predictors of disability and absenteeism in workers with non-specific low back pain: a longitudinal 15-month study. Appl Ergon. 2018;68:176–85. 29409632 10.1016/j.apergo.2017.11.011

[3] Wu A, March L, Zheng X, Huang J, Wang X, Zhao J, et al. Global low back pain prevalence and years lived with disability from 1990 to 2017: estimates from the global burden of disease study 2017. Ann Transl Med. 2020. 10.21037/atm.2020.02.175. 32355743 10.21037/atm.2020.02.175 PMC7186678

[4] Hartvigsen J, Hancock MJ, Kongsted A, Louw Q, Ferreira ML, Genevay S, et al. What low back pain is and why we need to pay attention. Lancet. 2018;391(10137):2356–67. 29573870 10.1016/S0140-6736(18)30480-X

[5] Parreira P, Maher CG, Steffens D, Hancock MJ, Ferreira ML. Risk factors for low back pain and sciatica: an umbrella review. Spine J. 2018;18(9):1715–21. 29792997 10.1016/j.spinee.2018.05.018

[6] Perera RS, Chen L, Ferreira ML, Arden NK, Radojčić MR, Kluzek S. Age-and sex-specific effects of obesity, metabolic syndrome and its components on back pain: the English longitudinal study of ageing. Joint Bone Spine. 2022;89(5):105366. 35227920 10.1016/j.jbspin.2022.105366

[7] Nindl BC, Billing DC, Drain JR, Beckner ME, Greeves J, Groeller H, et al. Perspectives on resilience for military readiness and preparedness: report of an international military physiology roundtable. J Sci Med Sport. 2018;21(11):1116–24. 29886134 10.1016/j.jsams.2018.05.005

[8] Yang Y, Liu S, Ling M, Ye C. Prevalence and potential risk factors for occupational low back pain among male military pilots: a study based on questionnaire and physical function assessment. Front Public Health. 2022;9:744601. 35059371 10.3389/fpubh.2021.744601 PMC8764305

[9] To D, Rezai M, Murnaghan K, Cancelliere C. Risk factors for low back pain in active military personnel: a systematic review. Chiropr Man Ther. 2021;29(1):52. 10.1186/s12998-021-00409-x PMC8719410 34969400

[10] Mattila VM, Kyröläinen H, Santtila M, Pihlajamäki H. Low back pain during military service predicts low back pain later in life. PLoS ONE. 2017;12(3):e0173568. 28282419 10.1371/journal.pone.0173568 PMC5345828

[11] Roy TC, Lopez HP, Piva SR. Loads worn by soldiers predict episodes of low back pain during deployment to Afghanistan. Spine. 2013;38(15):1310–7. 23532119 10.1097/BRS.0b013e31829265c4

[12] Chan EWM, Adnan R, Azmi R. Effectiveness of core stability training and dynamic stretching in rehabilitation of chronic low back pain patients. Malaysian J Mov Health Exerc. 2019;8(1):1–13.

[13] Sidiq M, Alenazi W, Kashoo FZ, Qasim M, Lopez MP, Ahmad M, et al. Prevalence of non-specific chronic low-back pain and risk factors among male soldiers in Saudi Arabia. PeerJ. 2021;9:e12249. 34721972 10.7717/peerj.12249 PMC8519176

[14] Hou Z, Shi J, Hong YE, Ni Z, Jun Y, Zheng L, et al. Prevalence of low back pain among soldiers at an army base. Chin Med J. 2013;126(4):679–82. 23422188

[15] George SZ, Childs JD, Teyhen DS, Wu SS, Wright AC, Dugan JL, et al. Rationale, design, and protocol for the prevention of low back pain in the military (POLM) trial (NCT00373009). BMC Musculoskelet Disord. 2007;8:1–11. 17868436 10.1186/1471-2474-8-92 PMC2034557

[16] George SZ, Childs JD, Teyhen DS, Wu SS, Wright AC, Dugan JL, et al. Brief psychosocial education, not core stabilization, reduced incidence of low back pain: results from the prevention of low back pain in the military (POLM) cluster randomized trial. BMC Med. 2011;9:1–12. 22126534 10.1186/1741-7015-9-128 PMC3286400

[17] Dietrich EJ, Leroux T, Santiago CF, Helgeson MD, Richard P, Koehlmoos TP. Assessing practice pattern differences in the treatment of acute low back pain in the United States military health system. BMC Health Serv Res. 2018;18:1–6. 30223830 10.1186/s12913-018-3525-8 PMC6142362

[18] Larson MJ, Adams RS, Ritter GA, Linton A, Williams TV, Saadoun M, et al. Associations of early treatments for low-back pain with military readiness outcomes. J Altern Complement Med. 2018;24(7):666–76. 29589956 10.1089/acm.2017.0290 PMC6065526

[19] Monnier A, Larsson H, Nero H, Djupsjöbacka M, Äng BO. A longitudinal observational study of back pain incidence, risk factors and occupational physical activity in Swedish marine trainees. BMJ Open. 2019;9(5):e025150. 31092646 10.1136/bmjopen-2018-025150 PMC6530317

[20] Xing W, Zhang Y, Yang Q, Wang X. Prevalence and risk factors of low back pain in military personnel: a systematic review. EFORT Open Rev. 2024;9(10):1002–12. 39360794 10.1530/EOR-22-0113 PMC11457804

[21] Chan EWM, Hamid MSA, Din FHM, Ahmad R, Nadzalan AM, Hafiz E. Prevalence and factors associated with low back pain among Malaysian army personnel stationed in Klang Valley. Biomed Hum Kinet. 2019;11(1):9–18. 10.2478/bhk-2019-0002.

[22] Gun BK, Banaag A, Khan M, Koehlmoos TP. Prevalence and risk factors for musculoskeletal back injury among US army personnel. Mil Med. 2022;187(7–8):e814–20. 34159385 10.1093/milmed/usab217

[23] Su CA, Kusin DJ, Li SQ, Ahn UM, Ahn NU. The association between body mass index and the prevalence, severity, and frequency of low back pain: data from the osteoarthritis initiative. Spine. 2018;43(12):848–52. 29462069 10.1097/BRS.0000000000002601

[24] Bedno SA, Jackson R, Feng X, Walton IL, Boivin MR, Cowan DN. Meta-analysis of cigarette smoking and musculoskeletal injuries in military training. Med Sci Sports Exerc. 2017;49(11):2191–7. 28614193 10.1249/MSS.0000000000001349

[25] Koszela K, Woldanska-Okonska M. The effect of smoking on back pain intensity in rehabilitated patients treated conservatively and surgically for discopathy. Ann Agric Environ Med 2021;28(1):179–82. 10.26444/aaem/123871 33775085

[26] Knox JB, Orchowski JR, Scher DL, Owens BD, Burks R, Belmont PJ Jr. Occupational driving as a risk factor for low back pain in active-duty military service members. Spine J. 2014;14(4):592–7. 23992937 10.1016/j.spinee.2013.06.029

[27] Hammed AI, Agbonlahor EI. Interdependence of marital status and clinical characteristics of morbidity with health-related quality of life among low back pain patients. Biomed Hum Kinet. 2016;8(1):159–64.

[28] Wei G, Li H, Wang B, Wu J, Wu F, Lin Z. A retrospective cross-sectional survey of non-specific lower back pain among a cohort of Chinese army soldiers. Int J Surg. 2018;56:288–93. 29933098 10.1016/j.ijsu.2018.06.023

[29] Ernat J, Knox J, Orchowski J, Owens B. Incidence and risk factors for acute low back pain in active duty infantry. Mil Med. 2012;177(11):1348–51. 23198512 10.7205/milmed-d-12-00183

[30] Bounds CL, Coppieters MW, Thomson HW, Larsen B, Evans K. Efficacy of conservative interventions for musculoskeletal conditions on pain and disability in active serving military personnel—a systematic review. Mil Med. 2024;189(1–2):e66-75. 36722165 10.1093/milmed/usac409 PMC10824481

