# Supplementary material for: Prevalence and risk factors of low back pain among Lithuanian national defense volunteer forces
Source: BMC Musculoskelet Disord. 2025 Oct 17;26:978. doi: 10.1186/s12891-025-09235-1 (PMC12535166; doi:10.1186/s12891-025-09235-1)
Supplement: Supplementary file 1 — Supplementary Material 1 [file 12891_2025_9235_MOESM1_ESM.pdf]

Questionnaire

1. What is your gender?
    - o Male
    - o Female
    - o Prefer not to disclose
2. What is your age? (please specify): _______
3. What is your level of education?
    - o Basic
    - o Secondary
    - o Higher
    - o Professional
    - o Other (please specify): _______
4. What is your height? (please specify): _______ cm.
5. What is your weight? (please specify): _______ kg.
6. What is your military rank? (please specify): _______
7. What is your marital status?
    - o Single
    - o Widowed
    - o Married
    - o Divorced
    - o Prefer not to disclose
8. How many years have you served in the military? (please specify): _______
9. Do you smoke?
    - o Yes
    - o No
10. How would you rate your overall health?
    - o Very good
    - o Good
    - o Average
    - o Poor
    - o Very poor
11. Do you experience stress?
    - o Yes
    - o No

12. How would you rate your stress level?
    - Low
    - Moderate
    - High
13. Have you ever experienced lower back pain?
    - Yes
    - No
14. Have you experienced lower back pain in the last 12 months?
    - Yes
    - No
15. Have you missed military service due to lower back pain in the last 12 months?
    - Yes
    - No
16. Are you currently experiencing lower back pain?
    - Yes
    - No
17. If you have experienced lower back pain in the last 12 months, how long did it last?
    - 1–7 days
    - 8–30 days
    - More than 30 days
    - Every day
18. Has your lower back pain lasted longer than three months?
    - Yes
    - No
19. If you have experienced lower back pain, did you seek medical attention?
    - Yes
    - No
20. Please rate your usual lower back pain on a numeric pain scale (1 = no pain, 10 = worst pain):

    1 2 3 4 5 6 7 8 9 10
21. Have you ever had an injury in the lower back region?
    - Yes
    - No
22. Does your lower back pain affect your military service?
    - Yes
    - No

23. Do you think your lower back pain might be related to military service conditions?
    - Yes
    - No
24. Have you received information about lower back pain and its prevention?
    - Yes
    - No
25. Where do you get information about lower back pain and its prevention? (Select all that apply)
    - Medical specialists
    - Workplace seminars
    - Internet
    - Friends
    - Other (please specify): _______
26. Have you ever been trained in proper ergonomic principles (e.g., posture and movement techniques) to prevent lower back pain?
    - Yes
    - No
